# Integrating host-microbiota metabolic networks: how aromatic amino acids shape immune homeostasis and affect disease progression

**DOI:** 10.1038/s41423-026-01435-6

**Published:** 2026-06-01

**Authors:** Yanan Zhang, Xinya Zhao, Haoran Wang, Zhuobiao Zhang, Hui Shi, Yuhao Wang, Shu Jeffrey Zhu

**Affiliations:** 1 Department of Veterinary Medicine, College of Animal Sciences, Zhejiang University, Hangzhou, Zhejiang China; 2 College of Veterinary Medicine, China Agricultural University, Beijing, China; 3 Zhejiang Provincial Key Laboratory of Pancreatic Disease of The First Affiliated Hospital, Institute of Translational Medicine, Zhejiang University School of Medicine, Hangzhou, Zhejiang China; 4 Department of Critical Care Medicine, Sir Run Run Shaw Hospital, Zhejiang University School of Medicine, Hangzhou, China

**Keywords:** Aromatic amino acid metabolites, Gut microbiota, Immunometabolism, Host‒microbe interactions, Immune regulation, Mechanisms of disease, Crohn's disease

## Abstract

Gut microbial metabolism is intimately linked to host immune homeostasis. Aromatic amino acids (AAAs) serve as substrates for both host and microbial enzymes, yielding a diverse array of metabolites that shape immune responses at local and systemic sites. In this review, an integrated framework is provided for understanding how AAA metabolites orchestrate immune cell function. The journey of AAAs from dietary intake through intestinal absorption and microbial utilization is traced, emphasizing the cooperative metabolic networks that generate immunomodulatory compounds. How these metabolites act on dendritic cells, macrophages, T cells, and B cells through membrane receptors, nuclear receptors, and epigenetic modifications to achieve cell‑type‑specific effects is subsequently examined. Drawing on recent discoveries, including cooperative microbial interactions in tryptophan metabolism, AAA metabolism is best understood as an integrated network rather than separate host and microbial compartments. How the dysregulation of these pathways contributes to inflammatory bowel disease and infectious diseases is further discussed, and emerging therapeutic strategies targeting the microbiota‑metabolite‑immune axis are highlighted. By synthesizing molecular mechanisms, cellular targets, and disease contexts, this review offers a conceptual roadmap for precision interventions that leverage the intricate metabolic connections between the host and microbiota.

## Introduction

The human gut harbors a complex and dynamic microbial ecosystem that profoundly influences host physiology, with the immune system representing one of the most intensely regulated domains of this host–microbe interplay [1, 2]. Disruption of these finely tuned interactions is now recognized as a key driver of diverse immune-mediated disorders, including inflammatory bowel disease, autoimmune conditions, allergic diseases, and altered susceptibility to infections [3, 4]. Understanding how microbial signals are decoded into specific immunological outcomes therefore holds both fundamental biological importance and translational promise.

Central to this communication are the vast arrays of bioactive metabolites generated by the gut microbiota. These small molecules traverse the intestinal epithelium and act as versatile messengers, influencing immune cell development, differentiation, and function through mechanisms that often converge on metabolic reprogramming, transcriptional regulation, and intracellular signaling cascades [5]. Among this diverse repertoire, metabolites derived from the aromatic amino acids (AAAs) tryptophan, phenylalanine, and tyrosine have attracted particular interest as potent modulators of immune responses. Although these amino acids have long been appreciated for their roles in protein synthesis and as neurotransmitter precursors [6], recent discoveries have revealed that their catabolism by both host and microbial enzymes produces a structurally diverse collection of signaling molecules that directly shape immune cell function through defined molecular pathways [7, 8].

What makes AAAs stand out among other amino acids in the context of host‒microbe interactions? Three unique features collectively position them as privileged molecular hubs at this interface. First, their aromatic side chains confer distinctive chemical properties that allow extensive enzymatic modification by both host and microbial enzymes, generating a wide array of signaling molecules, such as indole derivatives, phenolic compounds, and trace amines, that are not produced from aliphatic amino acids. Second, the shikimate pathway for de novo AAA synthesis is present in many commensal bacteria but completely absent in mammals, creating an obligate dependency of the host on microbial metabolism for certain AAA‑derived signals. Third, AAAs serve as direct precursors to key neurotransmitters (serotonin, dopamine, and trace amines) and to potent ligands of the aryl hydrocarbon receptor (AHR), thereby directly linking microbial metabolism to both neural and immune regulation. These characteristics explain why AAA metabolites have evolved as major information carriers in the bidirectional dialog between the gut microbiota and the host immune system.

In this review, an integrated framework is provided for understanding how AAA metabolites function as molecular bridges linking the gut microbiota to the host immune system. The journey of AAAs from dietary intake through intestinal absorption and microbial utilization is first traced, highlighting the sophisticated transport systems that ensure their availability and the intricate microbial metabolic networks that convert them into bioactive compounds. The immunomodulatory actions of AAA metabolites across distinct immune cell populations, including those of dendritic cells, macrophages, innate lymphoid cells, T cells, and B cells, with an emphasis on how these metabolites engage membrane receptors, nuclear receptors, and epigenetic machinery to achieve cell type-specific effects, are then systematically examined. Building on this mechanistic foundation, the contributions of AAA metabolic pathways to inflammatory bowel disease and infectious diseases are explored, illustrating how pathway dysregulation fuels pathogenesis and offering perspectives on therapeutic strategies. Throughout, recent advances from our own work and others that have uncovered previously unappreciated metabolic circuits and cooperative networks are integrated, and the review concludes by identifying key unanswered questions that will shape future inquiry.

A central theme of this review is the interconnected nature of AAA metabolism. Rather than treating host and microbial pathways as separate entities, substrate competition, metabolic cross‑feeding, and bidirectional regulatory loops are considered intertwined processes that collectively shape the availability and composition of immunomodulatory metabolites. This integrative perspective reflects a broader evolution in the field: moving from a focus on isolated microbial species and their individual products toward understanding how cooperative metabolic networks influence host physiology. Recent discoveries of cross-feeding interactions in tryptophan metabolism and their functional consequences for intestinal homeostasis exemplify this shift. By synthesizing the molecular mechanisms, cellular targets, and disease contexts of AAA metabolite-mediated immune regulation, this review aims to provide a conceptual roadmap for developing precision therapeutic strategies that leverage the microbiota–metabolite–immune axis.

## Host and gut microbiome crosstalk via aromatic amino acids

Tryptophan, phenylalanine, and tyrosine constitute the three aromatic amino acids (AAAs). Their characteristic aromatic side chains allow these amino acids to contribute not only to protein conformational stability but also to the regulation of host metabolism [9]. Tyrosine can be synthesized endogenously from phenylalanine, whereas tryptophan and phenylalanine must be obtained from the diet; all three therefore require coordinated intestinal digestion, absorption, and subsequent metabolic transformation to perform their biological functions [10–14].

### Intestinal transport of dietary AAAs

The absorption of dietary AAAs is markedly efficient in the small intestine, with ~90% of amino acids being taken up by the time luminal contents reach the ileum [15]. This efficiency relies on a spatially organized array of transporters expressed on the apical and basolateral membranes of enterocytes [16]. On the apical side, the sodium‑dependent transporter B^0^AT1 (Slc6a19) serves as the primary conduit for neutral amino acids, including all three AAAs [17]. Once internalized, AAAs exit the cell via basolateral transporters with distinct specificities. TAT1 (Slc16a10) selectively exports aromatic amino acids, LAT2 (Slc7a8) acts as an antiporter that exchanges AAAs for other neutral amino acids, and LAT1 (Slc7a5) contributes to tryptophan efflux [18, 19].

AAAs that escape small‑intestinal absorption (accounting for ~2–7% of dietary protein) enter the large intestine, where a distinct set of transporters handles their uptake. The apical transporter ATB^0,+^ (Slc6a14) efficiently transports both neutral and cationic amino acids, whereas the basolateral SNAT2 (Slc38a2) mediates the export of neutral amino acids [18]. The expression of these transporters is finely tuned along the intestinal axis and is modulated by diurnal rhythms, feeding status, and post‑translational modifications, ensuring that amino acid availability remains stable across various physiological states [18].

### Microbial engagement with intestinal AAAs

The colon is a metabolically active space where residual dietary protein, host‑derived endogenous substrates (such as mucins and exfoliated epithelial cells), and de novo synthesized microbial products converge to form an accessible amino acid pool [20]. The impact of the gut microbiota on AAA availability is strongly illustrated by comparisons between germ‑free (GF) and conventionally raised mice: plasma tryptophan and tyrosine levels are more than 1.4-fold greater in GF animals, whereas bacterial AAA-derived metabolites, including indoxyl sulfate, phenyl sulfate, *p*‑cresol sulfate, and phenylpropionylglycine, are present only in conventional mice [21]. Thus, the microbiota not only consumes host‑available AAAs but also actively contributes to the diversity of AAA‑related metabolites.

To compete for luminal AAAs, gut bacteria have evolved sophisticated uptake systems. *Escherichia coli* (*E. coli*), for instance, expresses multiple permeases with distinct affinities and specificities. Among them, Mtr functions as a high-affinity tryptophan permease, TnaB functions as a low-affinity tryptophan permease, TyrP functions as a tyrosine-specific permease, and PheP functions as a phenylalanine-specific permease. Additional systems, such as broad-spectrum AroP and ABC transporters, can also transport all three AAAs simultaneously [22, 23]. Beyond scavenging, certain commensals possess the shikimate pathway, enabling de novo AAA synthesis from chorismate. These bacteria can release newly synthesized AAAs into the lumen via efflux pumps such as YddG, thereby influencing the composition of the intestinal amino acid pool [24, 25]. Although the quantitative contribution of bacterial biosynthesis to total AAA availability remains to be precisely determined, it is clear that the gut microbiota acts as both a consumer and a producer of AAAs, thereby profoundly shaping the metabolic landscape of the intestine.

## Microbial metabolic pathways converting AAAs into bioactive signals

**Fig. 1:**
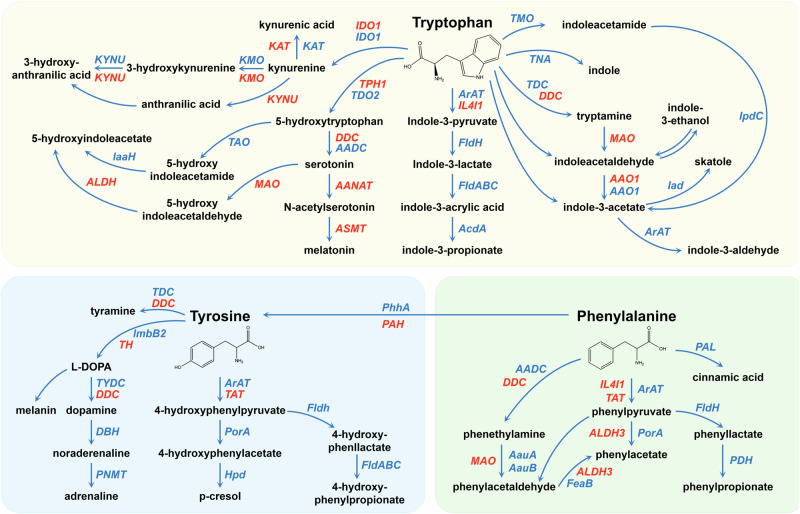
Integrated metabolic network of aromatic amino acid catabolism in the host-gut microbiota axis. The schematic illustrates the currently characterized metabolic pathways of aromatic amino acids within the gut microbiota and host cells. Microbial routes and the associated enzymes are depicted in blue, whereas host metabolic processes and their corresponding enzymes are shown in red

Once AAAs reach the colon, they become substrates for an extensive array of microbial enzymes that convert them into structurally diverse bioactive compounds (Fig. 1). These molecules serve as critical mediators of host–microbe communication, influencing immune function, metabolism, and tissue homeostasis. The following sections describe the major bacterial pathways for tryptophan, phenylalanine, and tyrosine, with an emphasis on the cooperative interactions that underpin metabolite production.

### Tryptophan metabolism and its cooperative networks

Bacterial Trp metabolism occurs through deamination, decarboxylation, and side‑chain cleavage, generating a cascade of indole‑containing intermediates. Initial products include indole, tryptamine, indole‑3‑acetamide, indole‑3‑acetaldehyde, and indole‑3‑pyruvate (IPyA), which are subsequently converted to terminal metabolites such as indole‑3‑acetic acid (IAA), indole‑3‑carboxaldehyde (IAld), and indole‑3‑propionic acid (IPA) [26, 27]. These pathways are not uniformly distributed across the microbiota. Firmicutes are major contributors to tryptamine and indole‑3‑pyruvate, whereas Proteobacteria specialize in indole‑3‑acetamide formation [26, 27].

Indole itself functions as an interbacterial signaling molecule, influencing sporulation, plasmid stability, drug resistance, biofilm formation, and virulence [28]. Other derivatives, such as indole‑3‑lactic acid (ILA) and indole‑3‑ethanol, exhibit antimicrobial properties that may help shape the microbial community composition [29, 30]. In contrast to these microbial pathways, the host metabolizes the majority of tryptophan through the kynurenine pathway (accounting for ~95% of catabolism) and the serotonin pathway, both of which are discussed in detail elsewhere [31].

A striking example of microbial cooperation is the production of IPA, a metabolite that has garnered attention for its role in intestinal epithelial repair. Our group recently showed that IPA-producing bacteria, such as *Peptostreptococcus russellii* (*P. russellii*), activate peroxisome proliferator-activated receptor α (PPARα) signaling in intestinal epithelial cells (IECs), upregulating the production of the ketogenic enzyme 3-hydroxy-3-methylglutaryl-CoA synthase 2 (HMGCS2) and increasing the production of β‑hydroxybutyrate (BHB). BHB then stimulates leucine-rich repeat-containing G-protein coupled receptor 5 (LGR5^+^) intestinal stem cells (ISCs), promoting epithelial regeneration [32]. Notably, IPA is often generated through cross‑feeding: ILA produced by *Blautia coccoides* (*B. coccoides*) is converted to IPA by *Clostridium sporogenes* (*C*. *sporogenes*) or *P. russellii*, and this conversion is essential for downstream effects on epithelial BHB synthesis [32]. Using an engineered *E. coli* strain expressing *B. coccoides* phenyllactate dehydrogenase (*fldH*), it was further demonstrated that both dietary tryptophan and bacterial *fldH* activity are required for ILA/IPA generation and subsequent mucosal healing [32]. These findings illustrate how tryptophan metabolism extends beyond single‑species activities to encompass integrated, community‑level networks.

### Shared pathways and emerging functions in phenylalanine and tyrosine metabolism

The metabolism of phenylalanine and tyrosine by gut bacteria produces a broad spectrum of phenolic compounds, with species‑specific contributions shaping the overall metabolite profile. Phenylalanine can be converted to phenylpyruvate, phenylacetate, and phenylpropionate. *Bacteroides ovatus* and *Bacteroides fragilis* preferentially generate phenylpyruvate, whereas *Bifidobacterium* species are major sources of phenylpropionate, and Clostridium species specialize in phenylacetate production [31]. Tyrosine gives rise to tyramine, 4‑hydroxyphenylpyruvate, 4‑hydroxyphenylacetate, and *p*‑cresol, with *Firmicutes* being the primary producers of tyramine and *Proteobacteria* contributing to 4‑hydroxyphenylpyruvate formation [33].

Beyond these well‑recognized pathways, recent studies have identified AAA-derived metabolites that exert systemic effects through immune and metabolic regulation. Phenyllactic acid (PLA), produced by *Lactobacillus* species that dominate the small intestine, protects against diet‑induced obesity during early life. In a model in which antibiotic exposure was combined with high-fat feeding, PLA increased peroxisome proliferator-activated receptor γ (PPARγ) expression in small intestinal epithelial cells, thereby modulating lipid metabolism and limiting obesity [34]. Similarly, 4-hydroxyphenylacetic acid (4HPAA) was negatively correlated with body fat accumulation in large-scale cohort analyses. In mice, 4HPAA and its analogs prevent high-fat diet-induced obesity by acting on the intestinal mucosa to regulate immune responses and lipid uptake, with innate lymphoid cells playing a key role [35]. These examples highlight how AAA metabolites can influence host metabolism by targeting both epithelial and immune pathways.

Phenethylamine, derived from bacterial decarboxylation of phenylalanine, has well‑documented neuromodulatory properties, while its hepatic conjugate phenylacetylglutamine promotes platelet activation and thrombosis, establishing it as a microbiota‑linked cardiovascular risk marker [33]. Tyramine, another bacterial product, modulates neurotransmission and immune function through trace amounts of amine‑associated receptors [36]. *p*‑Cresol has genotoxic potential in the colon, and its sulfated form, *p*‑cresyl sulfate, contributes to uremic toxicity in chronic kidney disease [33].

Cooperative interactions among bacteria further enrich the metabolic network. *C*. *sporogenes* initiates the reductive metabolism of phenylalanine and tyrosine via a phenylacetate dehydratase complex, yielding phenylpropionate and 4‑hydroxyphenylpropionic acid, products that support auxotrophic bacteria such as *Clostridioides difficile*, *Streptococcus pneumoniae*, and various *Lactobacillus* species. An alternative oxidative pathway, mediated by pyruvate‑ferredoxin oxidoreductase, produces phenylpropionate and 4‑hydroxyphenylacetate. Both routes operate in parallel, creating a metabolic support system that stabilizes the gut community [37].

Importantly, these pathways are dynamically regulated by nutrient availability. Under glucose-limited conditions, bacteria channel aromatic amino acids toward energy production; when glucose is abundant, the flux shifts toward the generation of bioactive molecules that influence community behavior [37]. This metabolic flexibility underscores the adaptive capacity of the gut microbiota and provides a mechanistic basis for its context-dependent effects on host physiology.

### Bidirectional regulation of host AAA metabolism by the microbiota

The interplay between the host and microbiota extends beyond substrate competition to include direct modulation of host metabolic enzymes. Bacterial metabolites such as propionate, butyrate, deoxycholate, tryptamine, and *p*‑aminobenzoate upregulate the expression of tryptophan hydroxylase 1 (Tph1), thereby influencing systemic serotonin levels [38]. Conversely, *Lachnospira*, *Clostridium*, *Roseburia*, and *Ruminococcus* species suppress indoleamine 2,3‑dioxygenase 1 (IDO1) expression through butyrate‑mediated inhibition of signal transducer and activator of transcription 1 (STAT1), an effect linked to histone deacetylase activity [39]. Serotonin metabolism is also developmentally regulated: neonatal gut bacteria, including *Rodentibacter heylii* and *Enterococcus gallinarum*, maximize serotonin availability by inducing high TPH1 expression while maintaining low monoamine oxidase A levels, a pattern that disappears in adulthood [40]. In addition, the microbiota influences AAA metabolism indirectly by modulating gastrointestinal motility. GF- or antibiotic-treated mice exhibit slowed intestinal transit and reduced enteric neuron density, alterations that affect AAA absorption and metabolism [41–44].

Collectively, these observations reveal that AAA metabolism is best understood as an integrated, bidirectional system. The gut microbiota does not merely consume or produce AAAs; it actively participates in a complex metabolic dialog with the host, shaping the composition of the intestinal metabolite pool through cooperative microbial networks and influencing host enzyme activity. AAA-derived metabolites also shape the gut microbial community itself, creating feedback loops that influence overall metabolite availability. Indole, for example, functions as an interbacterial signal that regulates sporulation, biofilm formation, and drug resistance in multiple species [28]. Tryptamine and *p*-cresol have been shown to inhibit the growth of certain commensals while promoting others, potentially altering community composition. Conversely, host-derived kynurenine can be utilized by some bacterial species as a carbon or nitrogen source, affecting their relative abundance [31–33]. These secondary effects, where metabolites derived from one bacterial species influence the growth and metabolic activity of others, add a further layer of regulatory nuance to the AAA metabolic network. This emerging picture, exemplified by the IPA‑ISC axis [32] and by the metabolic interactions surrounding phenylalanine and tyrosine, shifts the focus from individual species and isolated molecules toward network‑level interactions and their functional consequences for host physiology. A deeper understanding of these microbe‒metabolite‒microbe interactions will be essential for predicting the outcomes of therapeutic interventions targeting AAA metabolism and for translating this knowledge into therapeutic strategies that target the microbiota‒immune interface.

## Immune cell regulation by AAA metabolites

**Fig. 2:**
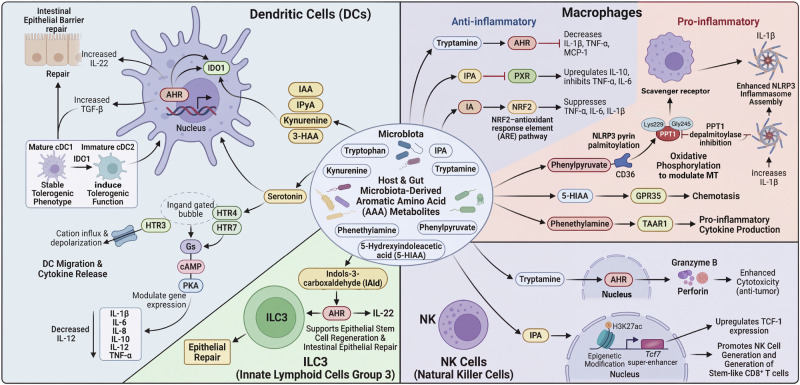
Multi-layered regulation of innate immunity by AAA metabolites. Host- and microbiota--derived aromatic amino acid (AAA) metabolites orchestrate innate immune responses through diverse signaling and epigenetic pathways. In dendritic cells (DCs), tryptophan-derived ligands (IAA, IPyA and 3-HAA) activate AHR to drive IL-22/TGF-β-mediated mucosal homeostasis. Kynurenine acts as an intercellular messenger: cDC1-derived kynurenine induces IDO1 expression in cDC2s via an AHR-dependent feedback loop, conferring a stable tolerogenic phenotype. Serotonin modulates DC function through HTR4/7-cAMP-PKA signaling to shift cytokine profiles (increases IL-1β/IL-8 and decreases IL-12/TNF-α) or via HTR3-mediated depolarization to increase migration. In macrophages, metabolites have different effects on polarization. Tryptamine, IPA, and IA suppress pro-inflammatory cytokines (TNF-α, IL-6, and IL-1β) through the AHR, PXR, and NRF2 pathways. Conversely, phenylpyruvate promotes NLRP3 inflammasome assembly by inhibiting PPT1-mediated depalmitoylation at Cys6, driving IL-1β production. Additionally, 5-HIAA and phenethylamine enhance chemotaxis and inflammation via GPR35 and TAAR1, respectively. With respect to ILC3s and NK cells, IAld activates AHR-IL-22 signaling in ILC3s to support epithelial repair. In NK cells, tryptamine enhances cytotoxicity (granzyme B, perforin) through AHR, whereas IPA promotes NK cell generation via H3K27ac-mediated upregulation of TCF-1. 3-HAA 3-hydroxyanthranilic acid, 5-HIAA 5-hydroxyindoleacetic acid, AHR aryl hydrocarbon receptor, cDC conventional dendritic cell, HTR 5-hydroxytryptamine receptor, IA indole-3-acrylic acid, IAA indole-3-acetic acid, IAld indole-3-carboxaldehyde, IDO1 indoleamine 2,3-dioxygenase 1, ILC3 group 3 innate lymphoid cell, IPA indole-3-propionic acid, IPyA indole-3-pyruvic acid, NK natural killer, NLRP3 NLR family pyrin domain containing 3, PPT1 palmitoyl-protein thioesterase 1, PXR pregnane X receptor, TAAR1 trace amine-associated receptor 1

**Fig. 3:**
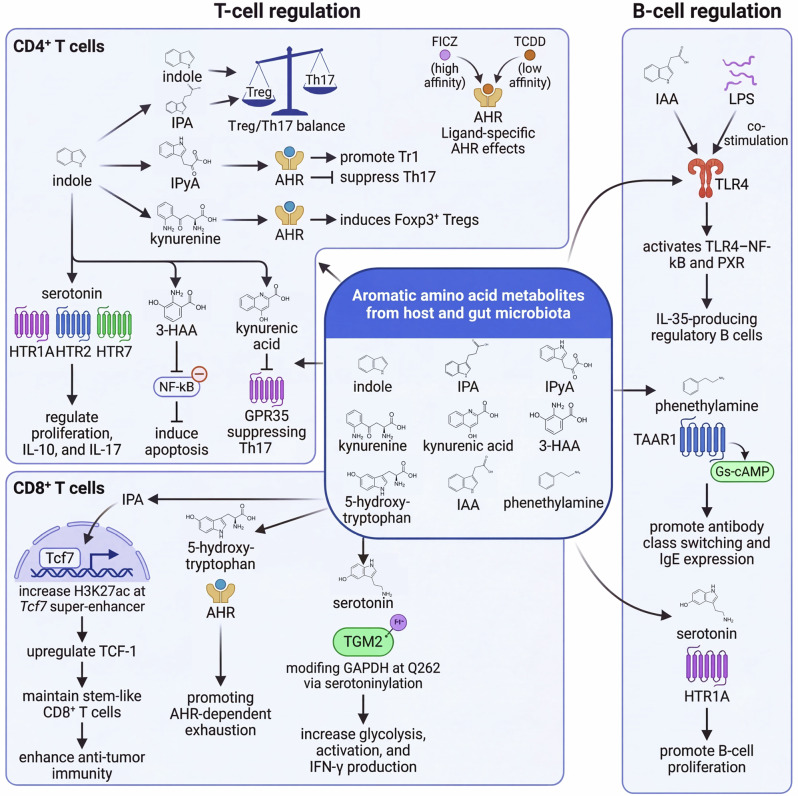
Adaptive immune cell regulation by host- and microbiota-derived aromatic amino acid metabolites. AAA metabolites orchestrate adaptive immunity through receptor dependent, transcriptional, and metabolic mechanisms. In CD4^+^ T cells, indole, IPA, IPyA, and kynurenine affect the Treg-Th17 balance, with ligand-specific AHR signaling further skewing lineage commitment toward either Th17 or Foxp3^+^ regulatory T cells. Serotonin modulates proliferation and cytokine production via HTR1A, HTR2, and HTR7, whereas 3-HAA induces apoptosis by inhibiting NF-κB, and kynurenic acid suppresses Th17 differentiation through GPR35. In CD8^+^ T cells, IPA promotes H3K27 acetylation at the *Tcf7* super-enhancer, increasing TCF-1 expression and sustaining stem-like, antitumor CD8^+^ T-cell states, whereas 5-hydroxytryptophan drives AHR-dependent exhaustion. Serotonin also enhances CD8^+^ T-cell activation via TGM2-mediated serotoninylation of GAPDH, which increases glycolysis and IFN-γ production. In B cells, IAA cooperates with LPS to activate TLR4-NF-κB and PXR signaling, driving IL-35-producing regulatory B cells; phenethylamine signals through TAAR1-Gs-cAMP to promote antibody class switching and IgE expression; and serotonin enhances B-cell proliferation via HTR1A. Collectively, these pathways position AAA metabolites as central integrators of microbiota–host communication in adaptive immunity. AAA aromatic amino acid, AHR aryl hydrocarbon receptor, GAPDH glyceraldehyde-3-phosphate dehydrogenase, HTR 5-hydroxytryptamine receptor, IAA indole-3-acetic acid, IFN-γ interferon-γ, IPA indole-3-propionic acid, IPyA indole-3-pyruvate, LPS lipopolysaccharide, 3-HAA 3-hydroxyanthranilic acid, PXR pregnane X receptor, TCF-1 T-cell factor-1, TGM2 transglutaminase 2, TLR4 Toll-like receptor 4

As detailed above, AAAs that escape host absorption are metabolized by the gut microbiota into a diverse array of bioactive molecules that serve as key messengers linking diet, microbiota, and host immunity. These metabolites act on distinct immune cell populations to orchestrate both innate (Fig. 2) and adaptive immune responses (Fig. 3). The following sections integrate the recognition mechanisms, signal transduction pathways, and functional outcomes for each immune cell type, providing a comprehensive view of how AAA metabolites shape immune regulation.

### Dendritic cells

Dendritic cells (DCs) play a central role in immune regulation and exhibit remarkable sensitivity to AAA metabolites. Multiple tryptophan-derived metabolites modulate DC function, with the aryl hydrocarbon receptor (AHR) pathway playing a pivotal role. In mouse bone marrow-derived dendritic cells, IAA activates AHR, markedly increasing the expression and secretion of interleukin‑22 (IL-22) [45], a cytokine critical for maintaining epithelial barrier integrity in the intestine. In mouse dendritic cells, IPyA similarly promotes a tolerogenic DC phenotype through AHR signaling, thereby influencing subsequent T-cell responses [46]. In mouse and human dendritic cells, kynurenine exerts complex regulatory effects: it induces indoleamine 2,3‑dioxygenase 1 (IDO1) expression via the transforming growth factor‑β (TGF-β) pathway while simultaneously activating AHR to promote TGF-β production, establishing a potential feedback loop that sustains immune tolerance under inflammatory conditions [47, 48]. 3-Hydroxyanthranilic acid (3-HAA) also activates AHR signaling, increasing TGF-β expression in DCs and further supporting their tolerogenic state [49].

In human monocyte-derived dendritic cells, serotonin signaling involves multiple receptor subtypes. These cells express 5‑hydroxytryptamine receptor 3 (HTR3), 5-hydroxytryptamine receptor 4 (HTR4), and 5-hydroxytryptamine receptor 7 (HTR7). HTR4 and HTR7 are coupled to stimulatory G proteins (Gs), leading to increased intracellular cyclic cAMP (cAMP) levels upon activation; this in turn activates protein kinase A (PKA), which modulates downstream gene expression, selectively enhancing the secretion of IL-1β and IL-8 while suppressing the production of IL-12 and tumor necrosis factor‑α (TNF-α) [50, 51]. More specifically, HTR4- and HTR7-mediated signaling upregulates the release of IL-1β, IL-6, IL-8, and IL-10, whereas HTR7 specifically inhibits the release of IL-12 and TNF-α [51]. HTR3 belongs to the ligand-gated ion channel family; its activation triggers rapid cation influx and membrane depolarization, contributing to DC migration and cytokine release [52].

Recent work from our group has provided new insights into how microbial metabolites regulate DC function. Using a combination of antibiotic-induced microbiota remodeling, metabolomic screening, intestinal organoids, GF mice, and gene-knockout models, we identified *Dubosiella newyorkensis* (and its human counterpart *Clostridium innocuum*) as a core functional bacterium that maintains intestinal mucosal immune homeostasis. Its metabolite L-lysine activates AHR in intestinal DCs, inducing IDO1 expression and driving the metabolic reprogramming of tryptophan toward kynurenine [53]. Kynurenine in turn activates AHR, establishing a positive-feedback AHR-IDO1-kynurenine metabolic loop. This circuit confers a stable tolerogenic phenotype on DCs, promoting regulatory T-cell differentiation and suppressing Th17 inflammatory responses.

Notably, kynurenine-mediated immune regulation extends beyond individual DCs. Gargaro and colleagues demonstrated that kynurenine functions as a metabolic messenger that transfers tolerogenic capacity between DC subsets [47]. Under steady-state conditions, only mature CCR7^+^ conventional DC subset type I (cDC1) cells express IDO1, and this expression depends on the transcription factor interferon regulatory 8 (IRF8). After lipopolysaccharide (LPS) stimulation, isolated immature cDC1s upregulate IDO1 expression and acquire tolerogenic activity, whereas conventional DC subset type 2 (cDC2) cells do not. However, when cDC2s are cocultured with mature cDC1s, L-kynurenine derived from cDC1s induces IDO1 expression and tolerogenic activity in cDC2s [47]. Thus, the immunoregulatory capacity of IDO1-expressing cDC1s is extended to cDC2s through the production of kynurenine, creating a metabolic axis for intercellular communication.

The physiological relevance of kynurenine as an AHR ligand remains a subject of debate. Some studies indicate that the concentrations required for kynurenine to activate AHR are greater than those typically present in the intestine or lymphoid tissues [27]. Nevertheless, in vivo phenomena such as the lysine-AHR-IDO1-kynurenine loop and kynurenine-mediated cDC1-cDC2 metabolic communication observed in our work and by others can be blocked by genetic or pharmacological interventions, suggesting that under specific microenvironments or in particular cell types that highly express kynurenine transporters, local kynurenine concentrations may reach thresholds sufficient for AHR activation. The possibility that kynurenine acts synergistically with other metabolites, such as 6-formylindolo[3,2-b]carbazole (FICZ), also warrants further investigation.

### Macrophages

Macrophages respond to AAA metabolites through a complex network of signaling pathways, with distinct metabolites regulating their inflammatory status and polarization via different mechanisms. Among tryptophan metabolites, in mouse bone marrow-derived macrophages, both IPA and indole-3-acrylic acid (IA) suppress proinflammatory cytokine production through distinct mechanisms: the former activates the pregnane X receptor (PXR) to upregulate IL-10 while inhibiting TNF-α and IL-6 [54], whereas the latter acts via the NF erythroid 2‑related factor 2 (NRF2)-antioxidant response element (ARE) pathway to suppress TNF-α, IL-6, and IL-1β expression [54]. Tryptamine inhibits the release of inflammatory mediators through AHR signaling, significantly reducing the expression of IL-1β, TNF-α, and monocyte chemoattractant protein‑1 (MCP‑1) [55]. The phenylalanine metabolite phenethylamine modulates M1 macrophage inflammation by regulating oxidative phosphorylation, illustrating the role of metabolic reprogramming in immune regulation [7]. The C‑C motif chemokine ligand 2 (CCL2)/C‑C chemokine receptor type 2 (CCR2) axis also contributes to this diversity [56], highlighting the diversity of regulatory mechanisms involved.

Some AAA metabolites exhibit proinflammatory potential. In human macrophages derived from diabetic foot ulcer patients, phenylpyruvate activates the NOD-like receptor thermal protein domain associated protein 3 (NLRP3) inflammasome, inducing IL-1β production [57]. In diabetic foot ulcers, increased phenylpyruvate enters macrophages via the CD36 scavenger receptor and binds to palmitoyl-protein thioesterase 1 (PPT1) at residues Lys229 and Gly245, inhibiting its depalmitoylase activity. This inhibition increases the palmitoylation of NLRP3 within its pyrin domain, enhancing inflammasome assembly and driving sustained inflammation [57]. 5-Hydroxyindoleacetic acid (5-HIAA) promotes macrophage chemotaxis through G protein‑coupled receptor 35 (GPR35) activation [58], whereas phenethylamine induces proinflammatory cytokine production in macrophages via trace amine-associated receptor 1 (TAAR1) signaling [59].

### Innate lymphoid cells and natural killer cells

Innate lymphoid cells (ILCs) and natural killer (NK) cells respond to tryptophan metabolites primarily through transcriptional and epigenetic mechanisms. In mouse group 3 innate lymphoid cells (ILC3s), IAld activates AHR signaling to promote IL-22 production, which in turn supports intestinal stem cell regeneration [60, 61]. In a *Citrobacter rodentium* infection model, IAld-induced IL-22 secretion from ILC3s enhanced intestinal epithelial repair [61]. Tryptamine enhances NK cell cytotoxicity against tumor cells via AHR activation, increasing the expression of granzyme B and perforin [62].

IPA regulates NK cells through epigenetic modification. In mouse tumor models, Jia and colleagues reported that IPA enhances H3K27 acetylation at the *Tcf7* superenhancer, upregulating T-cell factor‑1 (TCF-1) expression and promoting the differentiation of exhausted progenitor CD8^+^ T cells [63]. In the tumor microenvironment, this mechanism helps maintain stem-like CD8^+^ T-cell populations and enhances antitumor immune responses. Although studies directly investigating AAA metabolite regulation in ILCs and NK cells are relatively limited, existing evidence clearly implicates AHR signaling and epigenetic modification as central mechanisms, suggesting that microbial metabolites may influence innate lymphoid cell development and function through broader pathways.

### T cells

T cells are central targets of AAA metabolite-mediated adaptive immune regulation, with multiple signaling pathways converging to influence their differentiation, effector functions, and cytokine profiles.

In CD4^+^ T cells, distinct signaling cascades fine-tune differentiation outcomes. Multiple tryptophan metabolites, including indole and IPA, regulate the balance between regulatory T cells (Tregs) and Th17 cells through both AHR-dependent and AHR-independent pathways [64]. Notably, AHR activation has ligand-specific effects: while FICZ promotes Th17 differentiation, 2,3,7,8‑tetrachlorodibenzo‑p‑dioxin (TCDD) preferentially induces forkhead box P3 (Foxp3^+^) Tregs [65, 66], suggesting that different indole derivatives may have opposing effects on T-cell fate. IPyA similarly promotes Treg1 differentiation while suppressing Th17 responses through AHR signaling [46]. Kynurenine induces CD4^+^ T-cell differentiation toward Foxp3^+^ Tregs in an AHR-dependent manner [67, 68].

Serotonin signaling in T cells involves multiple receptor subtypes. T cells predominantly express HTR1A, HTR2, and HTR7, which are coupled to distinct G proteins and modulate intracellular cAMP levels, thereby influencing the production of cytokines such as IL-10 and IL-17 [69]. Specifically, HTR1A activation promotes T-cell proliferation, whereas HTR2-mediated signaling enhances IL-17 secretion [69]. 3-HAA induces T-apoptosis by inhibiting nuclear factor‑κB (NF-κB) [70], demonstrating that metabolites can control T-cell populations through both functional regulation and cell survival pathways. Kynurenic acid suppresses Th17 differentiation via GPR35 signaling [71], adding another layer to tryptophan metabolite-mediated T-cell regulation.

In CD8^+^ T cells, IPA enhances H3K27 acetylation at the *Tcf7* superenhancer, upregulating TCF-1 expression and promoting the differentiation of progenitor exhausted CD8^+^ T cells in a TCF-1-dependent manner [63]. In tumor models, this mechanism maintains stem-like CD8^+^ T-cell populations and enhances antitumor immunity. 5-Hydroxytryptophan influences CD8^+^ T-cell exhaustion through AHR signaling, suppressing T-cell function in the tumor microenvironment [72]. An additional layer of regulation comes from serotoninylation: this covalent modification, catalyzed by transglutaminase 2 (TGM2), attaches serotonin to the glutamine residues of target proteins. In human and mouse CD8^+^ T cells, serotoninylation of glyceraldehyde‑3‑phosphate dehydrogenase (GAPDH) at position Q262 promotes its cytoplasmic localization and enhances glycolytic metabolism, supporting T-cell activation and interferon‑γ (IFN-γ) production. Engineered chimeric antigen receptor (CAR) T cells with increased serotonin production exhibit enhanced therapeutic potential through this pathway [73].

### B cells

B cells exhibit diverse responses to AAA metabolites. In the presence of LPS, IAA induces the differentiation of IL-35-producing B cells through the coordinated activation of both the PXR pathway and the TLR4 pathway, an example of signal convergence in immune regulation [74]. Specifically, IAA and LPS costimulation activates NF-κB via TLR4 while also engaging in PXR-mediated transcriptional regulation, together driving B-cell differentiation toward IL-35-producing regulatory B cells [74]. Phenethylamine enhances IgE expression through TAAR1 signaling; as a G-protein-coupled receptor, TAAR1 couples to Gs in B cells, increasing cAMP levels and regulating antibody class switching [59]. Serotonin promotes B-cell proliferation via HTR1A activation, as evidenced by its ability to enhance the mitogenic response of B cells [75]. These observations illustrate how distinct receptor systems mediate the fine-tuned control of B-cell function by AAA metabolites.

### Concluding remarks on immune cell regulation

In summary, AAA metabolites orchestrate immune cell function with remarkable diversity and precision. Through recognition by membrane receptors (GPCRs, TLRs, and ion channels), activation of nuclear receptors (AHR and PXR), and induction of epigenetic modifications (histone acetylation and serotoninylation), these metabolites achieve multilayered regulation of immune cells. At the membrane receptor level, the GPCR family, which includes trace amine-associated receptors (TAARs), hydroxycarboxylic acid receptor 3 (HCA3), GPR35, and multiple serotonin receptors, couples to distinct G‑protein subtypes (Gs, Gi, Gq) to regulate second messengers such as cAMP and mitogen‑activated protein kinase (MAPK), initiating rapid signal transduction. Non‑GPCR receptors such as TLR4 and epidermal growth factor receptor (EGFR) expand the signaling repertoire by engaging pathways such as the NF‑κB and MAPK pathways. At the nuclear receptor level, AHR and PXR act as ligand‑dependent transcription factors that, following cellular uptake of metabolites, drive sustained transcriptional reprogramming. At the epigenetic level, serotoninylation and histone acetylation result in stable modifications to cellular function. Despite these advances, important questions remain: How do the same metabolite produce distinct effects in different cell types, and how do diverse signaling pathways integrate within specific immune microenvironments? Answering these questions will be crucial for understanding how microbial metabolites shape immune responses.

## Disease associations and therapeutic potential

**Fig. 4:**
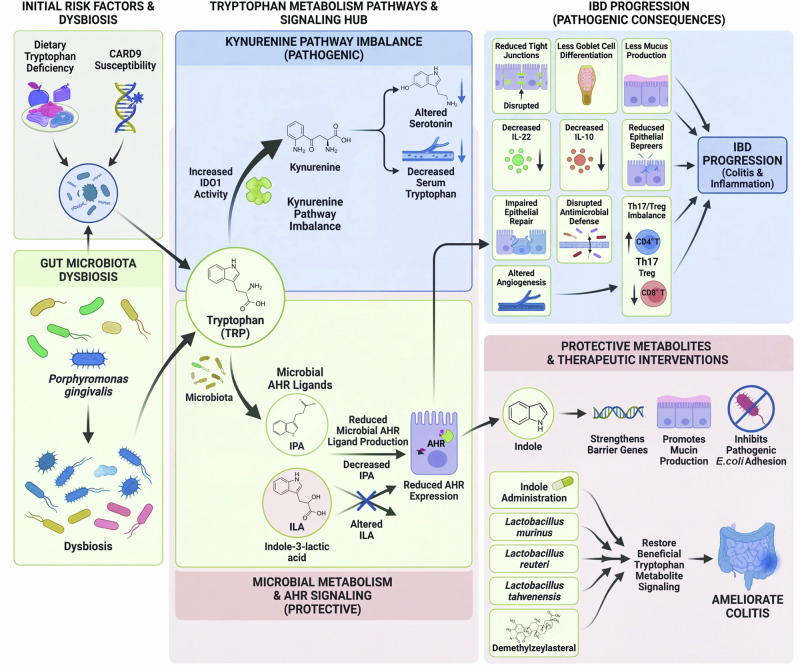
Disruption of tryptophan metabolism links gut microbiota dysbiosis to inflammatory bowel disease progression and identifies therapeutic avenues. Altered tryptophan metabolism represents a critical nexus between gut microbiota dysbiosis and inflammatory bowel disease (IBD). Dietary tryptophan insufficiency, *CARD9*-associated genetic susceptibility, and microbial dysbiosis collectively impair the conversion of tryptophan into microbiota-derived aryl hydrocarbon receptor (AHR) ligands, including protective indole metabolites such as indole-3-propionic acid (IPA) and indole-3-lactic acid (ILA). This impairment is accompanied by kynurenine pathway imbalance and increased indoleamine 2,3-dioxygenase 1 (IDO1) activity. Diminished AHR signaling compromises epithelial barrier integrity through multiple mechanisms: weakening of tight junctions, impaired goblet cell differentiation and mucus production, and reduced expression of IL-22 and IL-10. These defects collectively disrupt epithelial repair, antimicrobial defense, angiostatic regulation, and the Th17–Treg balance, thereby promoting intestinal inflammation and driving IBD progression. Conversely, the restoration of beneficial tryptophan metabolite signaling, achieved through indole administration, probiotic intervention (*Lactobacillus murinus*, *L. reuteri*, and *L. taiwanensis*), or pharmacological agents such as demethylzeylasteral, reinforces barrier-associated gene programs, enhances mucin production, inhibits pathogenic *E. coli* adhesion, and alleviates colitis. Collectively, these pathways establish microbiota--dependent tryptophan metabolism as a central determinant of intestinal homeostasis, disease susceptibility, and therapeutic responsiveness in IBD. AHR aryl hydrocarbon receptor, CARD9 caspase recruitment domain-containing protein 9, IBD inflammatory bowel disease, IDO1 indoleamine 2,3-dioxygenase 1, ILA indole-3-lactic acid, IPA indole-3-propionic acid, Th17 T helper 17 cell, Treg regulatory T cell

**Fig. 5:**
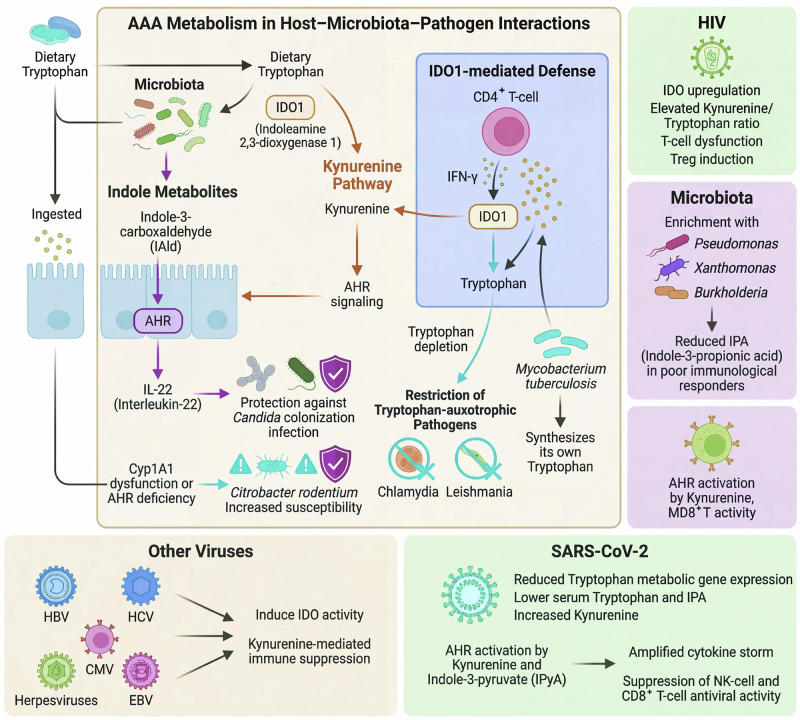
AAA metabolism in host-microbiota-pathogen interactions and infectious diseases. Tryptophan metabolism serves as a central regulatory axis in host defense against infectious diseases. Microbiota-derived indole metabolites, particularly IAld, activate AHR to drive IL-22 production, thereby reinforcing mucosal barrier function against fungal and bacterial pathogens such as *Candida* species and *Citrobacter rodentium*. Concurrently, host IDO1 directs tryptophan flux into the kynurenine pathway, restricting tryptophan-auxotrophic pathogens, including *Chlamydia* and *Leishmania*, whereas *Mycobacterium tuberculosis* circumvents this defense by endogenous tryptophan synthesis. In viral infections, particularly HIV and SARS-CoV-2, dysregulated IDO1-kynurenine metabolism is correlated with immune dysfunction, impaired antiviral immunity, and disease severity. Additional viruses, including hepatitis B virus (HBV), hepatitis C virus (HCV), herpesviruses, CMV, and Epstein–Barr virus (EBV), similarly engage IDO activation and kynurenine-mediated immune suppression. Collectively, these observations position AAA metabolism as a key metabolic interface that coordinates mucosal protection, pathogen restriction, and infection-associated immune dysregulation. AAA aromatic amino acid, AHR aryl hydrocarbon receptor, CMV cytomegalovirus, EBV Epstein–Barr virus, HBV hepatitis B virus, HCV hepatitis C virus, IAld indole-3-carboxaldehyde, IDO1 indoleamine 2,3-dioxygenase 1, IFN-γ interferon-γ, IL-10 interleukin-10, IL-22 interleukin-22, IPA indole-3-propionic acid, NK natural killer, Treg regulatory T cell

The preceding sections detail how AAA metabolites, through multilayered mechanisms involving membrane receptor signaling, nuclear receptor transcriptional regulation, and epigenetic modifications, precisely modulate the functions of dendritic cells, macrophages, T cells, and B cells. These mechanistic insights provide a foundation for understanding how these metabolites contribute to disease pathogenesis. Among the three AAAs, tryptophan metabolism has been most extensively studied; its metabolites perform central immunomodulatory functions through key nodes such as AHR and IDO1, and a wealth of translational research has been conducted in inflammatory bowel disease (IBD) (Fig. 4) and infectious diseases (Fig. 5) in recent years. Accordingly, the following sections focus on tryptophan as a representative AAA to systematically discuss the roles of AAA metabolite signaling pathways in disease processes and to explore their potential and challenges as therapeutic targets. A thorough understanding of these disease‑specific mechanisms is essential for developing precise immune‑modulatory strategies.

### Inflammatory bowel disease

Gut microbiota dysbiosis is firmly established in the pathogenesis of IBD, and alterations in tryptophan metabolism are considered critical links between microbial imbalance and intestinal inflammation (Fig. 5). Human clinical studies have revealed a significant negative correlation between serum tryptophan levels and IBD disease activity, with tryptophan deficiency promoting the onset and progression of IBD [76]. Concurrently, in IBD patients, the capacity of the gut microbiota to generate AHR ligands is impaired, a defect influenced by genetic factors such as the susceptibility gene caspase recruitment domain-containing protein 9 (CARD9) [77]. In line with these findings, AHR expression is consistently reduced in the intestinal tissues of IBD patients, particularly those with Crohn’s disease [78, 79]. Metabolite‑level analyses have shown decreased serum IPA in ulcerative colitis patients [80], whereas the levels of xanthurenic acid and kynurenic acid are inversely correlated with IBD severity; supplementation with these metabolites partially alleviates intestinal inflammation by activating AHR [81, 82]. In addition, IDO1 is overactivated both locally in the gut and systemically, with higher IDO1 activity observed in active IBD patients than in patients in remission, and serum tryptophan levels are negatively correlated with C‑reactive protein levels [76].

AHR plays pleiotropic roles in maintaining intestinal homeostasis, which explains why impaired AHR signaling contributes to IBD pathogenesis. At the barrier level, AHR signaling regulates tight junction proteins and promotes goblet cell differentiation and mucus production, collectively establishing a robust defense against pathogens [78, 83]. Indole reinforces this barrier by inducing the expression of genes that enhance mucosal barrier function and mucin production while inhibiting the adherence of pathogenic *E. coli* to epithelial cells [84]. Immunologically, AHR activation modulates the balance between T helper 17 (Th17) cells and regulatory T cells (Tregs), orchestrates innate and adaptive immune responses, and induces the expression of anti‑inflammatory cytokines such as interleukin‑10 (IL-10) and IL-22—factors essential for epithelial repair, mucus production, barrier integrity, and antimicrobial defense [79, 85]. AHR also contributes to intestinal vascular homeostasis, influencing angiogenesis and repair processes that are frequently disrupted in IBD [13].

The gut microbiota serves as a critical bridge between tryptophan metabolism and IBD pathogenesis. Insufficient dietary tryptophan alters the gut microbiota and impairs intestinal immune function [86]. Local tryptophan depletion can modulate bacterial proliferation [87]. Elevated intestinal serotonin levels promote the development of a colitogenic microbiota by stimulating bacterial growth, thereby exacerbating colitis [46]. Indole administration attenuates mucosal injury and inflammation in mice by reducing Bacteroidales levels and increasing Firmicutes and Clostridiales levels, changes that are crucial for maintaining intestinal homeostasis [88]. *Porphyromonas gingivalis* aggravates colitis by modulating the gut microbiota composition and linoleic acid levels, in turn affecting AHR activation and the Th17/Treg balance [89].

The pathogenic significance of these alterations has been further elucidated in animal models. *Ahr*‑deficient mice exhibit more severe colitis in T‑cell transfer or dextran sulfate sodium (DSS)-induced models, partly because of impaired IL-22 production [61, 90]. Card9-knockout mice, which carry an IBD susceptibility gene, harbor a dysbiotic microbiota that fails to convert tryptophan into AHR ligands, leading to reduced IL-22 release and increased susceptibility to DSS-induced colitis [77]. Supplementation with *Lactobacillus murinus*, *Lactobacillus reuteri*, or *Lactobacillus taiwanensis*—bacteria capable of inducing AHR ligand formation—ameliorates colitis in mice [77]. A recent study revealed that cecal levels of ILA were significantly elevated and positively correlated with symptoms in mice, whereas serum ILA levels were decreased and negatively correlated with ulcerative colitis symptoms; treatment with demethylzeylasteral restored both cecal and serum ILA levels and alleviated colitis [91].

The kynurenine pathway also contributes to IBD pathogenesis. *Ido1*-deficient mice are more susceptible to colitis, indicating that IDO1 acts as a negative regulator of intestinal inflammation. The pathological damage associated with IDO1 deficiency is partially attributable to the activation of proinflammatory cytokines and reduced numbers of colonic CD4^+^ Foxp3^+^ Tregs, although the specific metabolites and mechanisms involved remain to be elucidated [92]. As noted earlier, although kynurenine is widely reported as an AHR agonist, the concentrations required for AHR activation under physiological conditions are debated; thus, the precise role of kynurenine in IBD may involve more complex metabolic networks or indirect regulatory pathways.

### Infectious diseases

Tryptophan metabolites derived from the gut microbiota play important roles in protecting against mucosal infections (Fig. 5). In candidiasis caused by Candida species, the microbial production of AHR agonists such as IAld promotes IL-22 production, enhancing host resistance to fungal colonization [61]. Similarly, dysregulated degradation of AHR ligands due to *Cyp1a1* dysfunction or AHR deficiency increases susceptibility to Citrobacter rodentium infection [90, 93]. In both cases, restoring intestinal AHR activity reversed the heightened susceptibility, underscoring the importance of tryptophan metabolic homeostasis in infection defense.

Beyond the generation of AHR ligands, local tryptophan metabolism can serve as an adaptive component of host‒microbe interactions. Certain pathogens, such as the intracellular bacterium *Chlamydia* and the parasite *Leishmania*, are tryptophan auxotrophs—a vulnerability that CD4^+^ T cells exploit by upregulating IDO1 activity, diverting tryptophan to the kynurenine pathway and starving the pathogens. Some bacteria, however, have evolved counterstrategies; for example, *Mycobacterium tuberculosis* can synthesize its own tryptophan under stress conditions, thereby evading CD4^+^ T‑cell‑mediated defense [94]. Although these mechanisms have not yet been demonstrated in the intestinal environment, they offer valuable insights into the metabolic interplay between pathogens, commensals, and the host.

Tryptophan metabolism is also disrupted in viral infections. In HIV infection, IDO upregulation leads to an elevated kynurenine/tryptophan ratio that is correlated with disease progression. Kynurenine and its metabolites exacerbate T-cell dysfunction and promote Treg differentiation, further suppressing antiviral immune responses [95–98]. The gut microbiota contributes to this process; HIV‑infected patients exhibit enrichment of bacteria such as *Pseudomonas*, *Xanthomonas*, and *Burkholderia*, which encode enzymes of the kynurenine pathway, and *Pseudomonas fluorescens* has been shown to promote kynurenine production from tryptophan in vitro [99]. Moreover, serum levels of IPA are significantly lower in HIV patients who are poor immunological responders than in healthy individuals, suggesting an association between IPA levels and immune function [100].

In SARS‑CoV‑2 infection, hospitalized patients exhibit reduced expression of genes involved in tryptophan biosynthesis and metabolism, and serum levels of tryptophan and IPA are negatively correlated with proinflammatory mediator levels [101]. In vitro, IPA reduces interferon‑γ (IFN‑γ) production while promoting IL-22 and IL-10 generation, which is consistent with its role in modulating immune responses [101]. Notably, circulating kynurenine levels increase substantially during SARS‑CoV‑2 infection and are correlated with disease severity [102, 103]. Mechanistically, elevated kynurenine and indole‑3‑pyruvate activate AHR, contributing to immune dysregulation by triggering enhanced proinflammatory responses during the initial cytokine storm phase while simultaneously suppressing the antiviral activity of natural killer (NK) cells and CD8^+^ T cells [104, 105].

Other viruses, including hepatitis B virus (HBV), hepatitis C virus (HCV), herpesviruses, cytomegalovirus (CMV), and Epstein‒Barr virus (EBV), have also been linked to tryptophan metabolism. These viruses can affect the kynurenine pathway by inducing IDO activity [106], and the resulting accumulation of kynurenine pathway metabolites may further inhibit immune responses, for example, by inducing Tregs [106].

### Therapeutic strategies and future directions

The disease associations discussed above underscore the considerable potential but also the inherent complexity, of targeting AAA metabolite pathways for therapeutic intervention. In IBD, AHR signaling has a classic double-edged sword effect: although AHR activation can be protective by the induction of IL-22 and IL-10 [79, 107], a lack of ligand-specific control (FICZ promotes Th17 differentiation, whereas TCDD preferentially induces Tregs [65, 66]) may lead to opposing immunological outcomes. These observations suggest that successful therapeutic strategies must achieve ligand‑selective and cell‑type‑specific modulation rather than simply enhancing or suppressing AHR signaling. In infectious diseases, the IDO-kynurenine pathway presents another layer of complexity: moderate IDO activation can restrict the growth of tryptophan‑auxotrophic pathogens such as *Chlamydia*, whereas excessive activation may cause T‑cell dysfunction and immunosuppression, as observed in HIV and SARS‑CoV‑2 infections [97, 102, 103]. Thus, interventions targeting this pathway must strike a delicate balance between antimicrobial effects and the maintenance of immune homeostasis.

On the basis of these considerations, several directions for therapeutic development can be envisioned. First, direct supplementation with well‑defined immunomodulatory microbial metabolites such as IPA and IAld has been shown to have protective effects in animal models of IBD and infectious diseases [61, 80], but their pharmacokinetics, tissue targeting, and long‑term safety in humans require systematic evaluation. Second, modulating the microbial metabolic network through probiotics or engineered bacteria to restore the production of specific metabolites, for instance, by supplementation with IPA-producing *P. russellii* or ILA-producing *Lactobacillus* species, represents a more physiologically compatible intervention strategy [77, 91]. Third, the development of drugs targeting metabolite receptors such as AHR and PXR must carefully consider the functional selectivity conferred by ligand structural differences; selective AHR modulators that stably induce Treg differentiation without promoting Th17 responses are promising [65, 66]. Finally, patient stratification integrating metabolomic and metagenomic profiling will help identify those most likely to benefit from specific metabolite‑based interventions, enabling precision medicine approaches.

While tryptophan-derived metabolites have received the most attention, phenylalanine and tyrosine metabolites also hold diagnostic and therapeutic promise. PLA, produced by *Lactobacillus* species, has been shown to have protective effects against diet-induced obesity in early-life mouse models [34], and 4HPAA was shown in human cohort studies to be negatively correlated with body fat accumulation [35], suggesting its potential as a microbiome-targeted intervention for metabolic syndrome. In diagnostic applications, elevated circulating phenylacetylglutamine has emerged as a microbiota-linked biomarker for cardiovascular risk, and *p*-cresyl sulfate is a well-established uremic toxin whose levels predict disease progression in chronic kidney disease [33]. Moreover, bacterial decarboxylation products such as tyramine and phenethylamine, which act through TAARs, represent a class of metabolites with unexplored therapeutic potential for immune modulation [59]. Future studies should prioritize whether these metabolites can be harnessed as diagnostic markers, for example, through targeted metabolomics of patient plasma or feces, and whether precursor loading or probiotic strategies targeting phenylalanine/tyrosine pathways offer advantages over tryptophan-based interventions in specific disease contexts.

## Concluding remarks and perspectives

In summary, AAA metabolites act as molecular bridges connecting the immune system and the gut microbiota, orchestrating immune responses through multilayered signaling networks. These mechanisms span rapid signal transduction mediated by membrane receptors (G protein‑coupled receptors (GPCRs), Toll‑like receptors (TLRs), and others), sustained transcriptional reprogramming governed by nuclear receptors (AHR, PXR), and stable epigenetic modifications (histone acetylation, serotoninylation) that collectively shape the differentiation, activation, and effector functions of immune cells across different temporal and spatial scales. This review systematically synthesizes the regulatory mechanisms by which AAA metabolites act on dendritic cells, macrophages, innate lymphoid cells, T cells, and B cells, revealing a core principle: the same metabolite can elicit distinct functional outcomes in different cell types through the engagement of distinct receptors and signaling pathways.

Despite substantial progress, several fundamental questions remain. First, how do immune cells decode information encoded in the concentration, combination, and temporal dynamics of metabolites? Most studies to date have focused on single metabolites that act on single cell types; however, under physiological conditions, immune cells are exposed to complex, dynamic mixtures of metabolites. The mechanisms by which cells integrate such multiplex signals and distinguish nutritional cues from immunoregulatory cues are largely unknown. Addressing this question will require the development of new technologies capable of simultaneously measuring metabolite concentrations and signaling pathway activities at the single‑cell level, as well as the construction of in vitro coculture systems that recapitulate physiological complexity.

Second, how do metabolite signals interact and integrate with classical immune signals such as cytokines and T-cell receptor signals? The extensive crosstalk between AHR and pathways such as NF‑κB and NRF2 discussed in this review [108, 109] suggests that metabolite signaling is not an independent entity but is deeply embedded within the overall architecture of immune regulatory networks. However, the molecular details of this crosstalk, its cell‑type specificity, and its nodal role in the transition between inflammation and tolerance await systematic elucidation.

Third, what is the evolutionary logic underlying host‒microbe metabolic interactions? From an evolutionary perspective, why does the host tolerate the consumption of essential amino acids such as tryptophan by microbes to regulate its own immune responses? Conversely, why have microbes evolved such complex metabolic networks to influence host immunity? Understanding the mechanisms that maintain the homeostasis of this symbiotic relationship, as well as its dynamic changes across developmental stages (neonatal versus adult intestine) [40], holds both fundamental biological significance and translational relevance for immune‑related diseases.

Advances in single‑cell spatial metabolomics, metabolite imaging technologies, and tissue‑specific gene‑edited animal models will provide critical tools for addressing these questions [110, 111]. By integrating multi‑omics data with systems biology modeling, we may move from static maps of metabolite‒immune interactions toward the ability to predict the dynamic responses of the immune system to specific metabolite perturbations. Such a paradigm shift, from cataloging to mechanistic understanding to prediction, will ultimately propel the development of precision immune‑modulatory strategies based on metabolite regulation, opening new therapeutic avenues for IBD, infectious diseases, and other immune‑mediated disorders.

## References

[1] Kayama H, Okumura R, Takeda K. Interaction between the microbiota, epithelia, and immune cells in the intestine. Annu Rev Immunol. 2020;38:23–48. 32340570 10.1146/annurev-immunol-070119-115104

[2] Lavelle A, Sokol H. Gut microbiota-derived metabolites as key actors in inflammatory bowel disease. Nat Rev Gastroenterol Hepatol. 2020;17:223–37. 32076145 10.1038/s41575-019-0258-z

[3] Liu Y, Hou Y, Wang G, Zheng X, Hao H. Gut microbial metabolites of aromatic amino acids as signals in host-microbe interplay. Trends Endocrinol Metab. 2020;31:818–34. 32284282 10.1016/j.tem.2020.02.012

[4] Neavin DR, Liu D, Ray B, Weinshilboum RM. The role of the aryl hydrocarbon receptor (AHR) in immune and inflammatory diseases. Int J Mol Sci. 2018;19:3851. 30513921 10.3390/ijms19123851 PMC6321643

[5] O’Neill LAJ, Kishton RJ, Rathmell J. A guide to immunometabolism for immunologists. Nat Rev Immunol. 2016;16:553–65. 27396447 10.1038/nri.2016.70 PMC5001910

[6] Pogson CI, Knowles RG, Salter M. The control of aromatic amino acid catabolism and its relationship to neurotransmitter amine synthesis. Crit Rev Neurobiol. 1989;5:29–64. 2569940

[7] Zhang Q, Chen S, Guo Y, He F, Fu J, Ren W. Phenylalanine diminishes M1 macrophage inflammation. Sci China Life Sci. 2023;66:2862–76. 37243947 10.1007/s11427-022-2296-0

[8] Roager HM, Licht TR. Microbial tryptophan catabolites in health and disease. Nat Commun. 2018;9:3294. 30120222 10.1038/s41467-018-05470-4 PMC6098093

[9] Khemaissa S, Sagan S, Walrant A. Tryptophan, an Amino-Acid Endowed with Unique Properties and Its Many Roles in Membrane Proteins. Crystals. 2021;11:1032.

[10] Kumar V, Sharma A, Kaur R, Thukral AK, Bhardwaj R, Ahmad P. Differential distribution of amino acids in plants. Amino Acids. 2017;49:821–69. 28299478 10.1007/s00726-017-2401-x

[11] Tessari P, Lante A, Mosca G. Essential amino acids: Master regulators of nutrition and environmental footprint? Sci Rep. 2016;6:26074. 27221394 10.1038/srep26074 PMC4897092

[12] Rothhammer V, Quintana FJ. The aryl hydrocarbon receptor: An environmental sensor integrating immune responses in health and disease. Nat Rev Immunol. 2019;19:184–97. 30718831 10.1038/s41577-019-0125-8

[13] Stockinger B, Shah K, Wincent E. AHR in the intestinal microenvironment: Safeguarding barrier function. Nat Rev Gastroenterol Hepatol. 2021;18:559–70. 33742166 10.1038/s41575-021-00430-8 PMC7611426

[14] Yu K, Li Q, Sun X, Peng X, Tang Q, Chu H, et al. Bacterial indole-3-lactic acid affects epithelium-macrophage crosstalk to regulate intestinal homeostasis. Proc Natl Acad Sci USA. 2023;120:e2309032120. 37903267 10.1073/pnas.2309032120 PMC10636326

[15] Rowan AM, Moughan PJ, Wilson MN, Maher K, Tasman-Jones C. Comparison of the ileal and fecal digestibility of dietary amino acids in adult humans and evaluation of the pig as a model animal for digestion studies in man. Br J Nutr. 1994;71:29–42. 8312239 10.1079/bjn19940108

[16] Wang Y, Song W, Wang J, Wang T, Xiong X, Qi Z, et al. Single-cell transcriptome analysis reveals differential nutrient absorption functions in human intestine. J Exp Med. 2020;217:e20191130. 31753849 10.1084/jem.20191130 PMC7041720

[17] Jando J, Camargo SMR, Herzog B, Verrey F. Expression and regulation of the neutral amino acid transporter B0AT1 in rat small intestine. PLoS One. 2017;12:e0184845. 28915252 10.1371/journal.pone.0184845 PMC5600382

[18] Bröer S. Intestinal amino acid transport and metabolic health. Annu Rev Nutr. 2023;43:73–99. 37285555 10.1146/annurev-nutr-061121-094344

[19] Fotiadis D, Kanai Y, Palacín M. The SLC3 and SLC7 families of amino acid transporters. Mol Asp Med. 2013;34:139–58. 10.1016/j.mam.2012.10.007 23506863

[20] Neis EPJG, Dejong CHC, Rensen SS. The role of microbial amino acid metabolism in host metabolism. Nutrients. 2015;7:2930–46. 25894657 10.3390/nu7042930 PMC4425181

[21] Wikoff WR, Anfora AT, Liu J, Schultz PG, Lesley SA, Peters EC, et al. Metabolomics analysis reveals large effects of gut microflora on mammalian blood metabolites. Proc Natl Acad Sci USA. 2009;106:3698–703. 19234110 10.1073/pnas.0812874106 PMC2656143

[22] Burkovski A, Krämer R. Bacterial amino acid transport proteins: Occurrence, functions, and significance for biotechnological applications. Appl Microbiol Biotechnol. 2002;58:265–74. 11935175 10.1007/s00253-001-0869-4

[23] Steglich M, Hofmann JD, Helmecke J, Sikorski J, Spröer C, Riedel T, et al. Convergent loss of ABC transporter genes from clostridioides difficile genomes is associated with impaired tyrosine uptake and p-cresol production. Front Microbiol. 2018;9:901. 29867812 10.3389/fmicb.2018.00901 PMC5951980

[24] Mesnage R, Antoniou MN. Computational modeling provides insight into the effects of glyphosate on the shikimate pathway in the human gut microbiome. Curr Res Toxicol. 2020;1:25–33. 34345834 10.1016/j.crtox.2020.04.001 PMC8320642

[25] Doroshenko V, Airich L, Vitushkina M, Kolokolova A, Livshits V, Mashko S. YddG from escherichia coli promotes export of aromatic amino acids. FEMS Microbiol Lett. 2007;275:312–8. 17784858 10.1111/j.1574-6968.2007.00894.x

[26] Russell WR, Duncan SH, Scobbie L, Duncan G, Cantlay L, Calder AG, et al. Major phenylpropanoid-derived metabolites in the human gut can arise from microbial fermentation of protein. Mol Nutr Food Res. 2013;57:523–35. 23349065 10.1002/mnfr.201200594

[27] Hubbard TD, Murray IA, Perdew GH. Indole and tryptophan metabolism: Endogenous and dietary routes to ah receptor activation. Drug Metab Dispos. 2015;43:1522–35. 26041783 10.1124/dmd.115.064246 PMC4576673

[28] Lee J-H, Lee J. Indole as an intercellular signal in microbial communities. FEMS Microbiol Rev. 2010;34:426–44. 20070374 10.1111/j.1574-6976.2009.00204.x

[29] Narayanan TK, Rao GR. Beta-indoleethanol and beta-indolelactic acid production by candida species: Their antibacterial and autoantibiotic action. Antimicrob Agents Chemother. 1976;9:375–80. 1259397 10.1128/aac.9.3.375 PMC429538

[30] Bommarius B, Anyanful A, Izrayelit Y, Bhatt S, Cartwright E, Wang W, et al. A family of indoles regulate virulence and shiga toxin production in pathogenic *E. coli*. PLoS One. 2013;8:e54456. 23372726 10.1371/journal.pone.0054456 PMC3553163

[31] Dehhaghi M, Kazemi Shariat Panahi H, Guillemin GJ. Microorganisms, tryptophan metabolism, and kynurenine pathway: A complex interconnected loop influencing human health status. Int J Tryptophan Res. 2019;12:1178646919852996. 31258331 10.1177/1178646919852996 PMC6585246

[32] Zhang Y, Tu S, Shao X, Meng J, Zhang Z, Wei W, et al. Microbiota-derived IPA protects against colitis by regulating intestinal HMGCS2-mediated ketogenesis to facilitate mucosal healing. Nat Commun. 2026;17:2437. 41651833 10.1038/s41467-026-69341-z PMC12988232

[33] Al Hinai EA, Kullamethee P, Rowland IR, Swann J, Walton GE, Commane DM. Modeling the role of microbial p-cresol in colorectal genotoxicity. Gut Microbes. 2019;10:398–411. 30359553 10.1080/19490976.2018.1534514 PMC6546321

[34] Shelton CD, Sing E, Mo J, Shealy NG, Yoo W, Thomas J, et al. An early-life microbiota metabolite protects against obesity by regulating intestinal lipid metabolism. Cell Host Microbe. 2023;31:1604–1619.e10. 37794592 10.1016/j.chom.2023.09.002 PMC10593428

[35] Jiang Z, He L, Li D, Zhuo L, Chen L, Shi RQ, et al. Human gut microbial aromatic amino acid and related metabolites prevent obesity through intestinal immune control. Nat Metab. 2025;7:808–22. 40087408 10.1038/s42255-025-01246-5 PMC12021661

[36] Shimazu S, Miklya I. Pharmacological studies with endogenous enhancer substances: Beta-phenylethylamine, tryptamine, and their synthetic derivatives. Prog Neuropsychopharmacol Biol Psychiatry. 2004;28:421–7. 15093948 10.1016/j.pnpbp.2003.11.016

[37] Dodd D, Spitzer MH, Van Treuren W, Merrill BD, Hryckowian AJ, Higginbottom SK, et al. A gut bacterial pathway metabolizes aromatic amino acids into nine circulating metabolites. Nature. 2017;551:648–52. 29168502 10.1038/nature24661 PMC5850949

[38] Yano JM, Yu K, Donaldson GP, Shastri GG, Ann P, Ma L, et al. Indigenous bacteria from the gut microbiota regulate host serotonin biosynthesis. Cell. 2015;161:264–76. 25860609 10.1016/j.cell.2015.02.047 PMC4393509

[39] Martin-Gallausiaux C, Larraufie P, Jarry A, Béguet-Crespel F, Marinelli L, Ledue F, et al. Butyrate produced by commensal bacteria downregulates indolamine 2,3-dioxygenase 1 (IDO-1) expression via a dual mechanism in human intestinal epithelial cells. Front Immunol. 2018;9:2838. 30619249 10.3389/fimmu.2018.02838 PMC6297836

[40] Sanidad KZ, Rager SL, Carrow HC, Ananthanarayanan A, Callaghan R, Hart LR, et al. Gut bacteria-derived serotonin promotes immune tolerance in early life. Sci Immunol. 2024;9:eadj4775. 38489352 10.1126/sciimmunol.adj4775 PMC11328322

[41] Vicentini FA, Keenan CM, Wallace LE, Woods C, Cavin JB, Flockton AR, et al. Intestinal microbiota shapes gut physiology and regulates enteric neurons and glia. Microbiome. 2021;9:210. 34702353 10.1186/s40168-021-01165-z PMC8549243

[42] Anitha M, Vijay-Kumar M, Sitaraman SV, Gewirtz AT, Srinivasan S. Gut microbial products regulate murine gastrointestinal motility via toll-like receptor 4 signaling. Gastroenterology. 2012;143:1006–1016.e4. 22732731 10.1053/j.gastro.2012.06.034 PMC3458182

[43] Husebye E, Hellström PM, Sundler F, Chen J, Midtvedt T. Influence of microbial species on small intestinal myoelectric activity and transit in germ-free rats. Am J Physiol Gastrointest Liver Physiol. 2001;280:G368–380. 11171619 10.1152/ajpgi.2001.280.3.G368

[44] Zheng Z, Tang J, Hu Y, Zhang W. Role of gut microbiota-derived signals in the regulation of gastrointestinal motility. Front Med. 2022;9:961703. 10.3389/fmed.2022.961703 PMC9354785 35935766

[45] Ihekweazu FD, Engevik MA, Ruan W, Shi Z, Fultz R, Engevik KA, et al. *Bacteroides ovatus* promotes IL-22 production and reduces trinitrobenzene sulfonic acid-driven colonic inflammation. Am J Pathol. 2021;191:704–19. 33516788 10.1016/j.ajpath.2021.01.009 PMC8027925

[46] Aoki R, Aoki-Yoshida A, Suzuki C, Takayama Y. Indole-3-pyruvic acid, an aryl hydrocarbon receptor activator, suppresses experimental colitis in mice. J Immunol. 2018;201:3683–93. 30429284 10.4049/jimmunol.1701734

[47] Gargaro M, Scalisi G, Manni G, Briseño CG, Bagadia P, Durai V, et al. Indoleamine 2,3-dioxygenase 1 activation in mature cDC1 promotes tolerogenic education of inflammatory cDC2 via metabolic communication. Immunity. 2022;55:1032–1050.e14. 35704993 10.1016/j.immuni.2022.05.013 PMC9220322

[48] Pallotta MT, Orabona C, Volpi C, Vacca C, Belladonna ML, Bianchi R, et al. Indoleamine 2,3-dioxygenase is a signaling protein in long-term tolerance by dendritic cells. Nat Immunol. 2011;12:870–8. 21804557 10.1038/ni.2077

[49] Gargaro M, Vacca C, Massari S, Scalisi G, Manni G, Mondanelli G, et al. Engagement of nuclear coactivator 7 by 3-hydroxyanthranilic acid enhances activation of aryl hydrocarbon receptor in immunoregulatory dendritic cells. Front Immunol. 2019;10:1973. 31481962 10.3389/fimmu.2019.01973 PMC6710348

[50] Karmakar S, Lal G. Role of serotonin receptor signaling in cancer cells and anti-tumor immunity. Theranostics. 2021;11:5296–312. 33859748 10.7150/thno.55986 PMC8039959

[51] Idzko M, Panther E, Stratz C, Müller T, Bayer H, Zissel G, et al. The serotoninergic receptors of human dendritic cells: Identification and coupling to cytokine release. J Immunol. 2004;172:6011–9. 15128784 10.4049/jimmunol.172.10.6011

[52] Müller T, Dürk T, Blumenthal B, Grimm M, Cicko S, Panther E, et al. 5-hydroxytryptamine modulates migration, cytokine and chemokine release and T-cell priming capacity of dendritic cells in vitro and in vivo. PLoS One. 2009;4:e6453. 19649285 10.1371/journal.pone.0006453 PMC2714071

[53] Cui C, Feng X, Min Q, Lu C, Wen Z, Meng X, et al. A microbiota-IPA axis facilitates intestinal stem cell-mediated regeneration in colitis through a hopx-associated program. Nat Commun. 2026;17:1744147. 10.1038/s41467-026-70062-6 PMC13057028 41741429

[54] Wlodarska M, Luo C, Kolde R, d'Hennezel E, Annand JW, Heim CE, et al. Indoleacrylic acid produced by commensal peptostreptococcus species suppresses inflammation. Cell Host Microbe. 2017;22:25–37.e6. 28704649 10.1016/j.chom.2017.06.007 PMC5672633

[55] Krishnan S, Ding Y, Saedi N, Choi M, Sridharan GV, Sherr DH, et al. Gut microbiota-derived tryptophan metabolites modulate inflammatory response in hepatocytes and macrophages. Cell Rep. 2018;23:1099–111. 29694888 10.1016/j.celrep.2018.03.109 PMC6392449

[56] Xue C, Gu X, Zheng Q, Shi Q, Yuan X, Chu Q, et al. Effects of 3-HAA on HCC by regulating the heterogeneous macrophages-a scRNA-seq analysis. Adv Sci. 2023;10:e2207074. 10.1002/advs.202207074 PMC10238176 37013458

[57] Lv D, Cao X, Zhong L, Dong Y, Xu Z, Rong Y, et al. Targeting phenylpyruvate restrains excessive NLRP3 inflammasome activation and pathological inflammation in diabetic wound healing. Cell Rep Med. 2023;4:101129. 37480849 10.1016/j.xcrm.2023.101129 PMC10439185

[58] De Giovanni M, Tam H, Valet C, Xu Y, Looney MR, Cyster JG. GPR35 promotes neutrophil recruitment in response to serotonin metabolite 5-HIAA. Cell. 2022;185:815–830.e19. 35148838 10.1016/j.cell.2022.01.010 PMC9037118

[59] Moiseenko VI, Apryatina VA, Gainetdinov RR, Apryatin SA. Trace amine-associated receptors’ role in immune system functions. Biomedicines. 2024;12:893. 38672247 10.3390/biomedicines12040893 PMC11047934

[60] Hou Q, Ye L, Liu H, Huang L, Yang Q, Turner JR, et al. Lactobacillus accelerates ISCs regeneration to protect the integrity of intestinal mucosa through activation of STAT3 signaling pathway induced by LPLs secretion of IL-22. Cell Death Differ. 2018;25:1657–70. 29459771 10.1038/s41418-018-0070-2 PMC6143595

[61] Zelante T, Iannitti RG, Cunha C, De Luca A, Giovannini G, Pieraccini G, et al. Tryptophan catabolites from microbiota engage aryl hydrocarbon receptor and balance mucosal reactivity via interleukin-22. Immunity. 2013;39:372–85. 23973224 10.1016/j.immuni.2013.08.003

[62] Li S. Modulation of immunity by tryptophan microbial metabolites. Front Nutr. 2023;10:1209613. 10.3389/fnut.2023.1209613 PMC10382180 37521424

[63] Jia D, Wang Q, Qi Y, Jiang Y, He J, Lin Y, et al. Microbial metabolite enhances immunotherapy efficacy by modulating T cell stemness in pancancer. Cell. 2024;187:1651–1665.e21. 38490195 10.1016/j.cell.2024.02.022

[64] Kim WH, Lillehoj HS, Min W. Indole treatment alleviates intestinal tissue damage induced by chicken coccidiosis through activation of the aryl hydrocarbon receptor. Front Immunol. 2019;10:560. 30972060 10.3389/fimmu.2019.00560 PMC6443889

[65] Quintana FJ, Basso AS, Iglesias AH, Korn T, Farez MF, Bettelli E, et al. Control of T(reg) and T(H)17 cell differentiation by the aryl hydrocarbon receptor. Nature. 2008;453:65–71. 18362915 10.1038/nature06880

[66] Lee GR. Molecular mechanisms of T helper cell differentiation and functional specialization. Immune Netw. 2023;23:4 e4. 10.4110/in.2023.23.e4 PMC9995992 36911803

[67] Nguyen NT, Kimura A, Nakahama T, Chinen I, Masuda K, Nohara K, et al. Aryl hydrocarbon receptor negatively regulates dendritic cell immunogenicity via a kynurenine-dependent mechanism. Proc Natl Acad Sci USA. 2010;107:19961–6. 21041655 10.1073/pnas.1014465107 PMC2993339

[68] Campesato LF, Budhu S, Tchaicha J, Weng CH, Gigoux M, Cohen IJ, et al. Blockade of the AHR restricts a treg-macrophage suppressive axis induced by L-kynurenine. Nat Commun. 2020;11:4011. 32782249 10.1038/s41467-020-17750-z PMC7419300

[69] Wan M, Ding L, Wang D, Han J, Gao P. Serotonin: A potent immune cell modulator in autoimmune diseases. Front Immunol. 2020;11:186. 32117308 10.3389/fimmu.2020.00186 PMC7026253

[70] Hayashi T, Mo JH, Gong X, Rossetto C, Jang A, Beck L, et al. 3-hydroxyanthranilic acid inhibits PDK1 activation and suppresses experimental asthma by inducing T-a apoptosis. Proc Natl Acad Sci USA. 2007;104:18619–24. 18003900 10.1073/pnas.0709261104 PMC2141826

[71] Salimi Elizei S, Poormasjedi-Meibod M-S, Wang X, Kheirandish M, Ghahary A. Kynurenic acid downregulates IL-17/1 L-23 axis in vitro. Mol Cell Biochem. 2017;431:55–65. 28285360 10.1007/s11010-017-2975-3

[72] Liu Y, Zhou N, Zhou L, Wang J, Zhou Y, Zhang T, et al. IL-2 regulates tumor-reactive CD8+ T-cell exhaustion by activating the aryl hydrocarbon receptor. Nat Immunol. 2021;22:358–69. 33432230 10.1038/s41590-020-00850-9

[73] Wang X, Fu SQ, Yuan X, Yu F, Ji Q, Tang HW, et al. A GAPDH serotonylation system couples CD8+ T cell glycolytic metabolism to antitumor immunity. Mol Cell. 2024;84:760–775.e7. 38215751 10.1016/j.molcel.2023.12.015

[74] Su X, Zhang M, Qi H, Gao Y, Yang Y, Yun H, et al. Gut microbiota-derived metabolite 3-idoleacetic acid together with LPS induces IL-35+ B-cell generation. Microbiome. 2022;10:13. 35074011 10.1186/s40168-021-01205-8 PMC8785567

[75] Iken K, Chheng S, Fargin A, Goulet AC, Kouassi E. Serotonin upregulates mitogen-stimulated B lymphocyte proliferation through 5-HT1A receptors. Cell Immunol. 1995;163:1–9. 7758118 10.1006/cimm.1995.1092

[76] Nikolaus S, Schulte B, Al-Massad N, Thieme F, Schulte DM, Bethge J, et al. Increased tryptophan metabolism is associated with activity of inflammatory bowel diseases. Gastroenterology. 2017;153:1504–1516.e2. 28827067 10.1053/j.gastro.2017.08.028

[77] Lamas B, Richard ML, Leducq V, Pham HP, Michel ML, Da Costa G, et al. CARD9 impacts colitis by altering gut microbiota metabolism of tryptophan into aryl hydrocarbon receptor ligands. Nat Med. 2016;22:598–605. 27158904 10.1038/nm.4102 PMC5087285

[78] Marafini I, Monteleone I, Laudisi F, Monteleone G. Aryl hydrocarbon receptor signaling in the control of gut inflammation. Int J Mol Sci. 2024;25:4527. 38674118 10.3390/ijms25084527 PMC11050475

[79] Monteleone I, Rizzo A, Sarra M, Sica G, Sileri P, Biancone L, et al. Aryl hydrocarbon receptor-induced signals upregulate IL-22 production and inhibit inflammation in the gastrointestinal tract. Gastroenterology. 2011;141:237–248.e1. 21600206 10.1053/j.gastro.2011.04.007

[80] Alexeev EE, Lanis JM, Kao DJ, Campbell EL, Kelly CJ, Battista KD, et al. Microbiota-derived indole metabolites promote human and murine intestinal homeostasis through regulation of interleukin-10 receptor. Am J Pathol. 2018;188:1183–94. 29454749 10.1016/j.ajpath.2018.01.011 PMC5906738

[81] Pernomian L, Duarte-Silva M, de Barros Cardoso CR. The aryl hydrocarbon receptor (AHR) as a potential target for the control of intestinal inflammation: Insights from an immune and bacteria sensor receptor. Clin Rev Allerg Immunol. 2020;59:382–90. 10.1007/s12016-020-08789-3 32279195

[82] Michaudel C, Danne C, Agus A, Magniez A, Aucouturier A, Spatz M, et al. Rewiring the altered tryptophan metabolism as a novel therapeutic strategy in inflammatory bowel diseases. Gut. 2023;72:1296–307. 36270778 10.1136/gutjnl-2022-327337 PMC10314090

[83] Zhou X, Chakraborty D, Murray IA, Coslo D, Kehs Z, Vijay A, et al. Aryl hydrocarbon receptor activation coordinates mouse small intestinal epithelial cell programming. Lab Invest. 2023;103:100012. 37039146 10.1016/j.labinv.2022.100012

[84] Bansal T, Alaniz RC, Wood TK, Jayaraman A. The bacterial signal indole increases epithelial-cell tight-junction resistance and attenuates indicators of inflammation. Proc Natl Acad Sci USA. 2010;107:228–33. 19966295 10.1073/pnas.0906112107 PMC2806735

[85] Huang W, Rui K, Wang X, Peng N, Zhou W, Shi X, et al. The aryl hydrocarbon receptor in immune regulation and autoimmune pathogenesis. J Autoimmun. 2023;138:103049. 37229809 10.1016/j.jaut.2023.103049

[86] Gao J, Xu K, Liu H, Liu G, Bai M, Peng C, et al. Impact of the gut microbiota on intestinal immunity mediated by tryptophan metabolism. Front Cell Infect Microbiol. 2018;8:13. 29468141 10.3389/fcimb.2018.00013 PMC5808205

[87] Le Floc’h N, Otten W, Merlot E. Tryptophan metabolism, from nutrition to potential therapeutic applications. Amino Acids. 2011;41:1195–205. 20872026 10.1007/s00726-010-0752-7

[88] Whitfield-Cargile CM, Cohen ND, Chapkin RS, Weeks BR, Davidson LA, Goldsby JS, et al. The microbiota-derived metabolite indole decreases mucosal inflammation and injury in a murine model of NSAID enteropathy. Gut Microbes. 2016;7:246–61. 27007819 10.1080/19490976.2016.1156827 PMC4939928

[89] Jia L, Jiang Y, Wu L, Fu J, Du J, Luo Z, et al. *Porphyromonas gingivalis* aggravates colitis via a gut microbiota-linoleic acid metabolism-Th17/treg cell balance axis. Nat Commun. 2024;15:1617. 38388542 10.1038/s41467-024-45473-y PMC10883948

[90] Qiu J, Heller JJ, Guo X, Chen ZM, Fish K, Fu YX, et al. The aryl hydrocarbon receptor regulates gut immunity through modulation of innate lymphoid cells. Immunity. 2012;36:92–104. 22177117 10.1016/j.immuni.2011.11.011 PMC3268875

[91] Cheng Y, Yan D-M, Wu ZE, Zhu W-F, Li F. Integrated metabolomics and gut microbiome analysis reveal the role of demethylzeylasteral in alleviating ulcerative colitis in mice. J Proteome Res. 2025;24:3884–901. 40653681 10.1021/acs.jproteome.5c00035

[92] Takamatsu M, Hirata A, Ohtaki H, Hoshi M, Hatano Y, Tomita H, et al. IDO1 plays an immunosuppressive role in 2,4,6-trinitrobenzene sulfate-induced colitis in mice. J Immunol. 2013;191:3057–64. 23956437 10.4049/jimmunol.1203306

[93] Schiering C, Wincent E, Metidji A, Iseppon A, Li Y, Potocnik AJ, et al. Feedback control of AHR signaling regulates intestinal immunity. Nature. 2017;542:242–5. 28146477 10.1038/nature21080 PMC5302159

[94] Zhang YJ, Reddy MC, Ioerger TR, Rothchild AC, Dartois V, Schuster BM, et al. Tryptophan biosynthesis protects mycobacteria from CD4 T-cell-mediated killing. Cell. 2013;155:1296–308. 24315099 10.1016/j.cell.2013.10.045 PMC3902092

[95] Murray MF. Tryptophan depletion and HIV tryptophan depletion and HIV infection: A metabolic link to pathogenesis. Lancet Infect Dis. 2003;3:644–52. 14522263 10.1016/s1473-3099(03)00773-4

[96] Qi Q, Hua S, Clish CB, Scott JM, Hanna DB, Wang T, et al. Plasma tryptophan-kynurenine metabolites are altered in human immunodeficiency virus infection and associated with progression of carotid artery atherosclerosis. Clin Infect Dis. 2018;67:235–42. 29415228 10.1093/cid/ciy053 PMC6031054

[97] Schroecksnadel K, Zangerle R, Bellmann-Weiler R, Garimorth K, Weiss G, Fuchs D. Indoleamine-2,3-dioxygenase and other interferon-gamma-mediated pathways in patients with human immunodeficiency virus infection. Curr Drug Metab. 2007;8:225–36. 17430111 10.2174/138920007780362608

[98] Mándi Y, Vécsei L. The kynurenine system and immunoregulation. J Neural Transm. 2012;119:197–209. 21744051 10.1007/s00702-011-0681-y

[99] Vujkovic-Cvijin I, Dunham RM, Iwai S, Maher MC, Albright RG, Broadhurst MJ, et al. Dysbiosis of the gut microbiota is associated with HIV disease progression and tryptophan catabolism. Sci Transl Med. 2013;5:193ra91. 23843452 10.1126/scitranslmed.3006438 PMC4094294

[100] Feng A, Zhao H, Qiu C, Luo D, Wu H, Meng X, et al. Gut microbiota metabolites impact immunologic responses to antiretroviral therapy in HIV-infected men who have sex with men. Infect Dis Pover. 2025;14:21. 10.1186/s40249-025-01291-y PMC11917012 40098016

[101] Yao L, Devotta H, Li J, Lunjani N, Sadlier C, Lavelle A, et al. Dysrupted microbial tryptophan metabolism associates with SARS-CoV-2 acute inflammatory responses and long COVID. Gut Microbes. 2024;16:2429754. 39551951 10.1080/19490976.2024.2429754 PMC11581176

[102] Thomas T, Stefanoni D, Reisz JA, Nemkov T, Bertolone L, Francis RO, et al. COVID-19 infection alters kynurenine and fatty acid metabolism, correlating with IL-6 levels and renal status. JCI Insight. 2020;5:140327. 32559180 10.1172/jci.insight.140327 PMC7453907

[103] Michaelis S, Zelzer S, Schnedl WJ, Baranyi A, Meinitzer A, Enko D. Assessment of tryptophan and kynurenine as prognostic markers in patients with SARS-CoV-2. Clin Chim Acta. 2022;525:29–33. 34902346 10.1016/j.cca.2021.12.005 PMC8662911

[104] Anderson G, Carbone A, Mazzoccoli G. Tryptophan metabolites and aryl hydrocarbon receptor in severe acute respiratory syndrome, coronavirus-2 (SARS-CoV-2) pathophysiology. Int J Mol Sci. 2021;22:1597. 33562472 10.3390/ijms22041597 PMC7915649

[105] Li, M, Guo W, Dong Y, Wang X, Dai D, Liu X, et al. Elevated exhaustion levels of NK and CD8+ T cells as indicators for progression and prognosis of COVID-19 disease. Front Immunol. 2020;11:580237. 10.3389/fimmu.2020.580237 PMC7591707 33154753

[106] Mehraj V, Routy J-P. Tryptophan catabolism in chronic viral infections: Handling uninvited guests. Int J Trytophan Res. 2015;8:41–48. 10.4137/IJTR.S26862 PMC4527356 26309411

[107] Chen W, Pu A, Sheng B, Zhang Z, Li L, Liu Z, et al. Aryl hydrocarbon receptor activation modulates CD8αα+TCRαβ+ IELs and suppression of colitis manifestations in mice. Biomed Pharmacother. 2017;87:127–34. 28049094 10.1016/j.biopha.2016.12.061

[108] Vogel CF, Khan EM, Leung PS, Gershwin ME, Chang WL, Wu D, et al. Cross-talk between aryl hydrocarbon receptor and the inflammatory response: A role for nuclear factor-κB. J Biol Chem. 2014;289:1866–75. 24302727 10.1074/jbc.M113.505578 PMC3894361

[109] Dong H, Hao L, Zhang W, Zhong W, Guo W, Yue R, et al. Activation of AhR-NQO1 signaling pathway protects against alcohol-induced liver injury by improving redox balance. Cell Mol Gastroenterol Hepatol. 2021;12:793–811. 34082111 10.1016/j.jcmgh.2021.05.013 PMC8340139

[110] Hu T, Allam M, Cai S, Henderson W, Yueh B, Garipcan A, et al. Single-cell spatial metabolomics with cell-type specific protein profiling for tissue systems biology. Nat Commun. 2023;14:8260. 38086839 10.1038/s41467-023-43917-5 PMC10716522

[111] Ali A, Davidson S, Fraenkel E, Gilmore I, Hankemeier T, Kirwan JA, et al. Single cell metabolism: Current and future trends. Metabolomics. 2022;18:77. 36181583 10.1007/s11306-022-01934-3 PMC10063251

